# Identifying Genetic Signatures of Natural Selection Using Pooled Population Sequencing in *Picea abies*

**DOI:** 10.1534/g3.116.028753

**Published:** 2016-05-02

**Authors:** Jun Chen, Thomas Källman, Xiao-Fei Ma, Giusi Zaina, Michele Morgante, Martin Lascoux

**Affiliations:** * Department of Ecology and Genetics, Evolutionary Biology Centre and Science for Life Laboratory, Uppsala University, 75236 Sweden; † Department of Agricultural, Food, Environmental and Animal Sciences, University of Udine, 33100 Italy

**Keywords:** pooled sequencing, *F_ST_*, allele frequencies, local adaptation

## Abstract

The joint inference of selection and past demography remain a costly and demanding task. We used next generation sequencing of two pools of 48 Norway spruce mother trees, one corresponding to the Fennoscandian domain, and the other to the Alpine domain, to assess nucleotide polymorphism at 88 nuclear genes. These genes are candidate genes for phenological traits, and most belong to the photoperiod pathway. Estimates of population genetic summary statistics from the pooled data are similar to previous estimates, suggesting that pooled sequencing is reliable. The nonsynonymous SNPs tended to have both lower frequency differences and lower *F_ST_* values between the two domains than silent ones. These results suggest the presence of purifying selection. The divergence between the two domains based on synonymous changes was around 5 million yr, a time similar to a recent phylogenetic estimate of 6 million yr, but much larger than earlier estimates based on isozymes. Two approaches, one of them novel and that considers both *F_ST_* and difference in allele frequencies between the two domains, were used to identify SNPs potentially under diversifying selection. SNPs from around 20 genes were detected, including genes previously identified as main target for selection, such as *PaPRR3* and *PaGI*.

Identifying loci under selection and inferring past species history remain two of the main challenges in evolutionary biology. To a large extent, this is due to the difficulty of teasing apart the effects of selection and demography at the genetic level, and to the dearth of methods to estimate the effect of these two processes simultaneously (Li *et al.* 2012; Tiffin and Ross-Ibarra 2014). This is especially true for methods based on the detection of outliers from a null demographic model since an incorrect demographic model can lead to a large number of false positives or false negatives (Huber *et al.* 2014; Tiffin and Ross-Ibarra 2014). The difficulty is further compounded by the fact that selection can act at different spatial scales, and detection of local selection can simply be missed if one uses a sample of individuals drawn indiscriminately from a large population. Conversely, selection can leave a signature in some genes at the species range level, but no detectable signature at a more local scale, and vice versa. This is what is suggested by our recent studies in Norway spruce (*Picea abies*) and Siberian spruce (*P. obovata*), two Eurasian conifer species with a joint distribution extending from Norway eastwards to the Sea of Okhotsk. We have identified loci putatively under selection in two types of studies. First, using populations located along latitudinal clines, we combined tests of association to environmental variables, detection of *F_ST_* outliers and gene expression studies to detect evidence of local adaptation in genes related to variation in phenology, a trait known to exhibit strong latitudinal variation (Ekberg *et al.* 1979; Savolainen *et al.* 2007). In both species, two photoperiod pathway related genes, *PaFTL2* (*P. abies* Flowering Locus T like 2) and *PaGI* (*P. abies* Gigantea), appear to have been under local selection (Chen *et al.* 2012a, 2014). Second, using individuals sampled across the range of Norway spruce, we looked for departure from neutrality under different demographic models in 19 genes from the photoperiodic pathway. Only the circadian clock gene *PaPRR3* (*P. abies* Pseudo Response Regulator 3) deviated consistently from neutrality for all tested demographic scenarios, and the signature of selection observed at *PaPRR3*, a positive Tajima’s *D* value, indicative of an excess of common variants, was suggestive of local selection, at least at broad geographic scale (Källman *et al.* 2014). However, no evidence of selection at *PaPRR3* was detected in the aforementioned analyses of clines, which considered a more local or finer spatial scale.

In comparison to these previous studies, which either sampled rather densely along a cline, or used a scattered sampling across the whole natural range, the present study is based on an intermediate sampling strategy. The current study also differs in its resequencing approach. Here, we use next generation sequencing of two pools of individuals to assess polymorphism at 88 nuclear genes. Pooled sequencing reduces costs, and, if analyzed carefully, can provide reliable estimates of allele frequencies (Boitard *et al.* 2012; Futschik and Schlotterer 2010; Lynch *et al.* 2014). These polymorphism data are used to (i) infer the divergence time and migration rate between the two domains, using silent sites; and (ii) test for the presence of both purifying and adaptive selection in those genes. The two pools of individuals were sampled in the two main phylogeographic domains of Norway spruce, the Alpine domain, and the Fennoscandian domain (Figure 1). These two domains are today geographically separated and genetically differentiated (Heuertz *et al.* 2006; Lagercrantz and Ryman 1990), especially for mitochondrial DNA markers (Lockwood *et al.* 2013; Tollefsrud *et al.* 2009; Tsuda *et al.* 2016). The strong divergence observed at mtDNA markers, and the polyphyly *of P. abies* for mtDNA even led Lockwood *et al.* (2013) to suggest that the two domains may deserve to be considered as two different species. However, divergence between the two domains is not exceptionally high for 22 allozymes (Lagercrantz and Ryman 1990), and 16 nuclear genes (Heuertz *et al.* 2006), and certainly lower than the divergence from Siberian spruce (*P. obovata*) at both types of markers (Tsuda *et al.* 2016). With 88 nuclear loci the present dataset provides an opportunity to obtain a better estimate of divergence time and migration between the two domains compared to earlier studies. We also show that both purifying and directional selection have shaped polymorphism at some of the genes related to the photoperiodic pathway.

**Figure 1:**
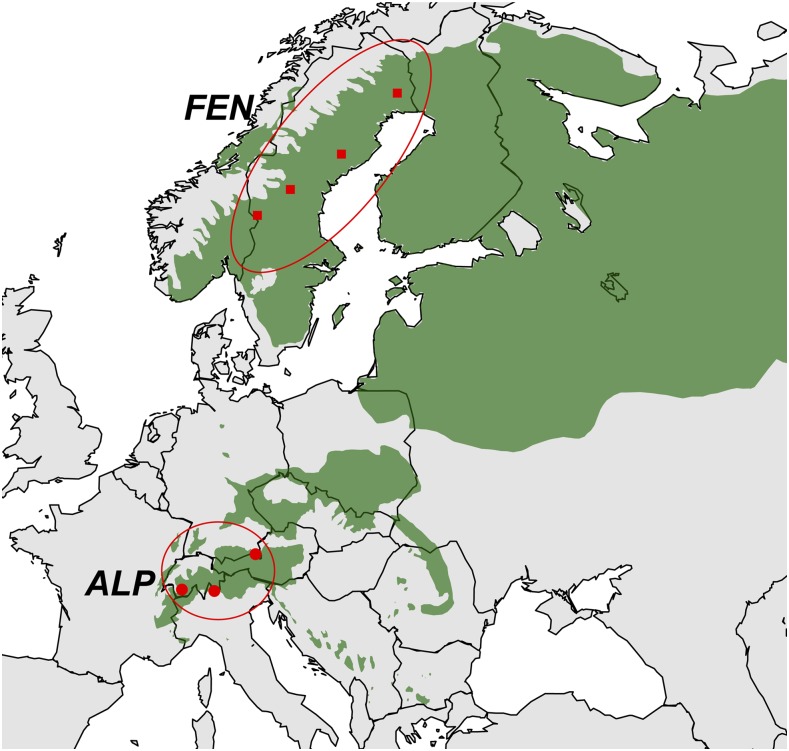
Map of population samples included in this study. The areas shaded in green are the distribution of Norway spruce (www.euforgen.org). Squares and dots represent the populations in FEN and ALP domains, respectively.

## Materials and Methods

### Sampling and sequencing

We germinated seeds from 48 maternal trees sampled in four Fennoscandian populations (coming from latitudes ranging from 61°N to 67°N), and from 48 maternal trees sampled in three Alpine populations (coming from latitudes ranging from 46°N to 47°N) (Table 1 and Figure 1). Diploid DNA was extracted from needles individually, using a DNeasy Plant Mini Kit (Qiagen, Germantown, MD). We PCR-amplified 88 gene fragments using the proofreading enzyme Phusion (Finnzymes, Espoo, Finland). We selected genes in two ways: (i) 30 of them come from our resequencing efforts; they were chosen since they are homologous to *Arabidopsis* or *Populus* genes that belong to the photoperiodic pathway, and, in particular, to the circadian clock; (ii) 58 of them originate from a previous unpublished microarray expression study. They had a rapid response to day length changes, and were highly divergent in gene expression during the bud set period (T. Källman *et al.*, unpublished data; Paper I in Källman 2009). Gene sequences and their best BLASTN hits in the Norway spruce genome have been deposited in Dryad (see *Data availability*). We blasted the reference sequences against the *P. abies* and *P. glauca* genomes and found them to be unique. We controlled for paralogs in two ways. First, we checked the gel bands in the lab. We also Sanger-sequenced genes using the megagametophytes of two individuals from the same origin as the pooled samples, and this did not suggest the presence of paralogs. Second, a BLAST search of reference sequence against *P. abies* and *P. glauca* genomes produced significant similarity differences between best hit and second hit. The bias introduced by possible paralogs depends on the percentage of paralogs distributed in individuals of both pools, and the percentage of paralog reads mapped/unmapped to the reference. This remains a difficult problem in nonmodel organisms whose genome is incomplete since exhaustive search is impossible for homolog/paralog/gene duplication.

**Table 1:** Sampling information

Pool domain	Code	No. Ind	Country	Latitude (°N)	Longitude (°E)	Altitude (m)
Fennoscandian domain (FEN)	SE-67	12	Sweden	66.58	22.70	140
	SE-64	12	Sweden	64.08	18.74	270
	SE-62	12	Sweden	62.63	15.12	351
	SE-61	12	Sweden	61.57	12.78	550
Alpine domain (ALP)	GE-47	20	Germany	47.68	12.65	1492
	CH-46	15	Switzerland	46.23	7.43	1286
	IT-46	13	Italy	46.17	9.73	553

Amplification products of all genes were diluted to a concentration of 10 ng/μl and mixed in equal proportions. Individuals from the different populations were pooled into two sequencing libraries corresponding to the Fennoscandian and Alpine domains, respectively. Mixed DNA samples were sheared into fragments of size between 150–200 bp using Bioruptor. Paired-end libraries were prepared and sequenced using next generation sequencing (NGS) technology on Illumina HiSeq2000 platform by Istituto di Genomica Applicata (IGA), Udine (Italy), with the read length equal to 109 bp. In order to identify errors arising from sequencing, two sequencing replicates were run for both pooled libraries. The Sanger-sequenced genes using the megagametophytes of two individuals from the same origin as the pooled samples were also used as references for short-read alignment.

### Read mapping and SNP calling

We trimmed sequencing reads using the program Trimmomatic v. 0.32 (Bolger *et al.* 2014), retaining only high-quality sequence pairs where each pair was at least 34 bp long after trimming. Read alignment was performed using a two-step SNP-tolerant mapping protocol. First, the trimmed reads were aligned to reference Sanger sequences using GSNAP (Wu and Nacu 2010; Wu and Watanabe 2005) with default settings for genomic mapping. Aligned reads were filtered with a mapping quality ≥ 20. We only kept those properly paired reads that were mapped to a unique position in the same gene (*i.e.*, concordant unique read pairs). Indel-realignment was performed using the Genome Analysis Toolkit (DePristo *et al.* 2011). We identified single nucleotide polymorphisms (SNPs) with Sanger-sequenced references in all sequencing libraries using samtools’ mpileup (Li *et al.* 2009). Missing sites in reference sequences were corrected by BLAST (Zhang *et al.* 2000) against the Spruce Genome Project (Nystedt *et al.* 2013), as well as the aligned sequence in both pools before the next step. In the second step, we used identified variants to perform SNP-tolerant read alignment using GSNAP. In order to have a uniform number of reads per gene, reads of each library were subjected to two steps of downsampling before and after aligning to references. We randomly picked reads without replacement to generate a down-sampled library of size equal to the smallest library, which contains 1,807,545 read pairs. We then repeated the quality filtering and indel-realignment as in the first step. Before SNP calling, we performed a further downsampling of aligned reads in each gene to reduce possible bias in frequency estimation caused by uneven distribution of read depth across gene body, as well as different amplification cycles among genes. We randomly picked 100 reads on both strands without replacement that covered the first base at 5′ end of the gene, and recalculated read depth in each position. We then kept downsampling at the next base toward the 3′ end until the number of reads at all positions reached 100 on both strands. Thus, in total, we expected the mean depth at each position to be around 200. At positions with a depth lower than 200, all reads were retained. Finally, SNP identification and allele frequency estimation were performed in all sequencing libraries using CRISP, a multi-sample variant caller for high-throughput pooled sequence data (Bansal 2010). To minimize the number of possible sequencing errors, the inference of an allele and its frequency was considered to be reliable only if: (i) the allele was supported by both sequencing replicates, and (ii) minor alleles could be identified in at least two reads in each replicate, or five reads in total across the two pools. Allele frequencies were polarized by comparing to the *P. glauca* homolog (Birol *et al.* 2013). Since we expected the minimum allele frequency to be at least one in 96, we considered rare alleles of frequency < 0.01 (which is also the 5% tail of frequency distribution of all alleles) to be biased estimates of unbalanced mixture of amplification products or possible mismatches introduced during the amplification and/or sequencing processes. However, we did not simply exclude them from our study since those estimates still represented sampling probability of rare alleles. Instead, we performed our analyses on the total dataset, as well as on subsets of SNPs with different frequency cutoffs. Two frequency cutoffs were used: ≥ 0.01 and ≥ 0.1.

### Nucleotide diversity and population divergence

We estimated pairwise nucleotide diversity (π) (Nei and Li 1979), Tajima’s *D* (Tajima 1989), and Wright’s fixation index *F*_ST_ (Wright 1931) values for each locus based on estimates of allele frequencies using custom R scripts. We chose Hudson’s estimator of *F*_ST_ (Hudson *et al.* 1992; Keinan *et al.* 2007) for each SNP, and an overall *F*_ST_ across all SNPs as an estimate of population divergence using the formulae derived by Bhatia *et al.* (2013):

$$F_{\text{ST}}=\frac{{\left(p_{1}-p_{2}\right)}^{2}-\frac{p_{1}\left(1-p_{1}\right)}{n_{1}-1}-\frac{p_{2}\left(1-p_{2}\right)}{n_{2}-1}}{p_{1}\left(1-p_{2}\right)+p_{2}\left(1-p_{1}\right)}$$ (1)

where *n*_1_, *n*_2_ and *p*_1_, *p*_2_ are the sample size and allele frequencies in the two samples, respectively. Hudson’s estimator is a better choice for pairs of populations since it has been shown to be more robust to sample size difference than other estimators, and *F*_ST_ is not systematically overestimated. To combine information across SNPs, two methods were used: we took the average of individual SNP *F*_ST_ values, or we calculated the ratio of the expectations of the denominator and numerator across all SNPs. The first estimator is more sensitive to the presence of SNPs with low minimum allele frequency (MAF), and the second is dominated by SNPs that are on average more polymorphic (Jackson *et al.* 2014).

### Inference of population demographic history

**Figure 2:**
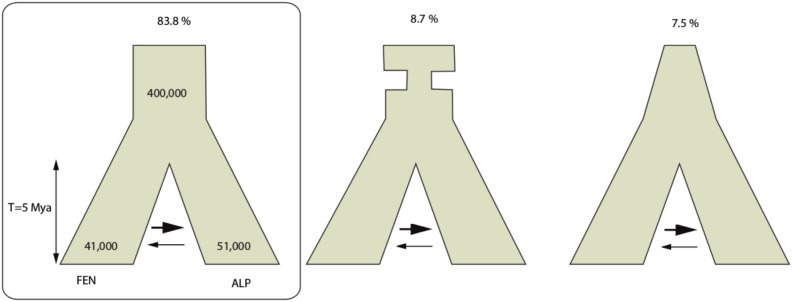
Models analyzed with fastsimcoal2. From left to right, constant ancestral population size, bottlenecked ancestral population, and expanding ancestral population. The constant ancestral population size model is the most likely as indicated by the percentage of bootstraps supporting each of the three models given above each tree. Figures within each branch are estimates of the effective population sizes of the descendent and ancestral populations.

To infer the population divergence history we used the program fastsimcoal2, (ver. 2.5.2.8), which implements a maximum likelihood method based on composite likelihoods of the two-dimensional joint site frequency spectra (SFS) (Excoffier *et al.* 2013). We considered three demographic models. All three models start with an ancestral population (*N*_0_) that split into two descendant populations with effective population size *N*_FEN_ and *N*_ALP_, respectively, at time *T* before present. Asymmetrical migration is allowed between the two descendant populations after divergence with a number of migrants equal to *M* = 2*Nm*, where *N* is the effective population size of one of the two descendant populations, and *m* is the migration rate that can differ for each descendant population. Three different demographic models were applied to the ancestral population: (i) constant population size; (ii) population expansion with a growth rate α; and (iii) bottleneck (Figure 2). In the latter, to reduce the complexity, we assumed that a bottleneck size of 0.001N_0_ occurred *T*_BOT_ generations ago, and lasted for 100 generations. We used all silent SNPs to calculate the site frequency spectrum, and projected it on a 10 × 10 grid. Maximum likelihood estimates of the model parameters were obtained using sequential Markov coalescent simulations, and an extension of the EM algorithm where each parameter of the model is maximized in turn, keeping the other parameters at their last estimated value (see Excoffier *et al.* 2013 for details). To estimate the confidence intervals around parameter estimates, we generated 1000 bootstrap datasets by binomial-sampling of alleles with the observed frequencies for all loci. Model comparisons were carried out using Akaike’s weight of evidence, as suggested by Excoffier *et al.* (2013). To assess the performance of the models, we applied a multinomial comparison between expected and observed SFS by calculating Anscombe residuals (Gutenkunst *et al.* 2009).

### SNP divergence analysis based on F_ST_ and allele frequency difference

In this analysis, we combined Hudson’s *F*_ST_ and allele frequency difference (AFD) as a distance measurement of SNP divergence between populations. By comparing to the expected distribution of the most likely demographic scenario, we identified outliers with higher divergence as the clue of either diversifying selection.

We first dispersed all SNPs on orthogonal coordinates of *F*_ST_ and AFD. The distance of each SNP to the origin (*F*_ST_ = 0, AFD = 0) was calculated as a new summary statistics for SNP divergence distance (div*D*) that combines *F*_ST_ and difference in allele frequency (AFD):

$$\text{div}D=\sqrt{F_{\text{ST}}{}^{2}+{\text{AFD}}^{2}}$$ (2)

We then dispersed SNPs on a third dimension by grouping them into nonsynonymous or silent (noncoding and synonymous) sites using a variable transformation based on factor analysis of mixed data (FAMD). We applied FAMD to our data using functions implemented in the R package ‘FactoMineR’ (Le *et al.* 2008). Divergence distance for a given gene was calculated by averaging values at SNPs within that gene.

To identify candidate SNPs under diversifying selection, we calculated SNP distance distribution from the simulation datasets: namely, we first generated 1000 expected SFS under the most likely demographic history using parameters estimated by 1000 bootstrap runs. For each expected SFS, we drew the same number of SNPs, and calculated the expected allele frequencies and *F*_ST_ values. Simulated SNPs were also randomly grouped as if they belonged to the same genes as in the observed dataset. For each simulated dataset, we then calculated div*D* for SNPs and genes and calculated the 95%, 97.5%, and 99% envelopes based on the ranked SNP distance to the origin. We used the maximum values of these three envelopes of the 1000 bootstrap runs as the cutoffs for outlier selection at 5%, 2.5%, and 1% significance levels.

### F_ST_ outliers

*F*_ST_ has been used extensively as a powerful statistic to identify loci under natural selection (*e.g.*, Beaumont and Balding 2004; Beaumont and Nichols 1996; Foll and Gaggiotti 2008). Compared to neutral SNPs, outlier SNPs with higher *F*_ST_ values could indicate the presence of diversifying selection, while lower *F*_ST_ values could hint at balancing selection. We performed the *F*_ST_ outlier test implemented in the program BayeScan version 2.1, which assumes that allele frequencies follow a multinomial Dirichlet distribution that arises under a wide range of demographic models (Foll and Gaggiotti 2008). To account for the difference in numbers of putative neutral and selected loci, we chose a prior odds ratio of 10, which should be conservative in our case. We performed 20 pilot runs with 5000 iterations and 500,000 iterations MCMC, with an additional 50,000 iterations as burn-in. We calculated Q-values (Storey 2003) as posterior probabilities corrected for multiple tests to estimate the likelihood of the model with selection compared to the neutral model.

### Data availability

All short-read libraries have been deposited in NCBI Sequence Read Archive (SRA) under accession number PRJNA272968. The reference sequences, gene annotation, SNPs, as well as custom Perl and R scripts used in this study have been deposited in Dryad http://dx.doi.org/10.5061/dryad.c5250.

## Results

### Sampling and sequencing

We isolated diploid DNA from 48 individuals drawn from populations sampled in the Fennoscandian domain (FEN) at latitudes ranging from 61 to 67°N, and 48 individuals drawn from populations of the Alpine domain (ALP) with latitudes ranging from 46°N to 47°N (Figure 1 and Table 1). We selected 88 different gene fragments covering in total 111,297 bp. On average, because of the filtering each base was covered by approximately 200 reads (Table 2 and Supplemental Material, Figure S1), and no significant difference was detected between the two pools (two-sample Kolmogorov-Smirnov test *p*-value = 0.99).

**Table 2:** Summary of aligned reads and number of alleles identified in both pooled population data sets

	FEN Domain		ALP Domain	
	runA	runB	runA	runB
No. reads	3,615,090	11,063,898	7,108,922	9,804,776
No. reads after 1st downsampling	3,615,090	3,615,090	3,615,090	3,615,090
No. reads aligned	2,492,388	2,215,690	2,485,110	2,231,850
No. reads aligned after 2nd downsampling	218,968	214,958	218,306	214,650
No. reads/base averaged across all genes*^a^*	221 (52–451)	205 (62–436)	221 (53–447)	206 (63–436)

a Mean and 95% confidence intervals are shown.

### Site frequency spectrum and nucleotide diversity

**Figure 3:**
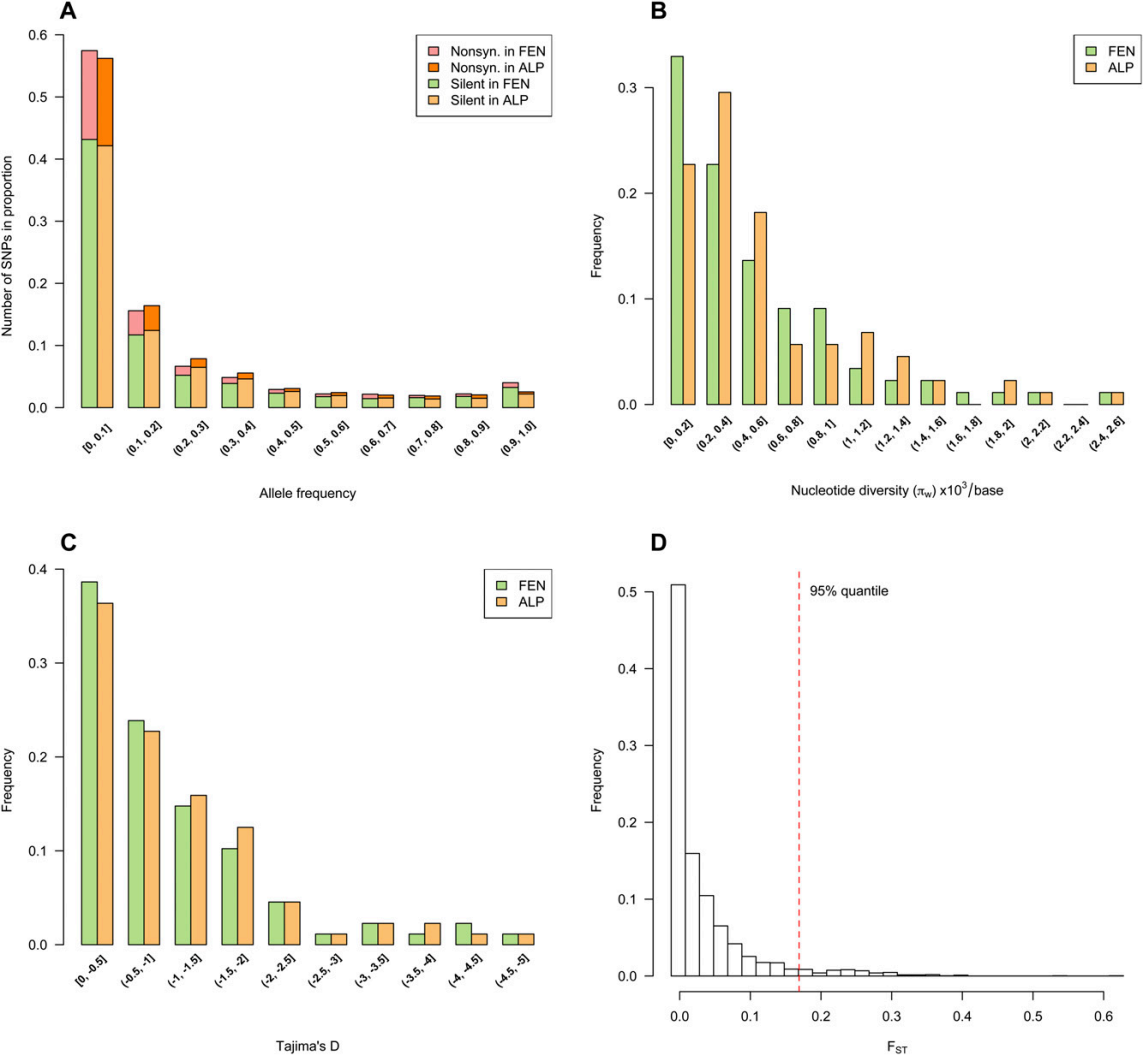
Distributions of summary statistics calculated based on all SNPs in both pools. (A) Site frequency spectra on silent and nonsynonymous SNPs; (B) Nucleotide diversity (π*_w_*); (C) Tajima’s *D*; (D) *F*_ST_.

In total, we identified 2791 SNPs from the 88 gene fragments; 2436 SNPs were shared between the two pools, and 60 and 295 SNPs were private to the FEN and ALP pools, respectively. The private SNPs tended to have a low frequency in the pool where they were found (the mean frequency of private SNPs in the FEN and ALP domains were 0.06 and 0.1, respectively). A total of 1600 SNPs as located in noncoding regions, and 546 SNPs were synonymous; 632 SNPs caused amino acid replacement (Figure 3A). We also found 11 premature stop-codons in open reading frames, and two mutations from stop-codons to amino acids. The site frequency spectra of both pools deviated from neutrality, showing an excess of minor alleles at low frequencies (< 0.1), possibly indicating population expansion after the two pools diverged.

On average, we observed a higher number of segregating sites per gene in the ALP dataset (mean *S* = 31) than in the FEN dataset (mean *S* = 28). We noticed slight difference in nucleotide diversity between the two datasets using paired Wilcoxon Rank Sum test (mean π = 0.0056 and 0.0051, for ALP and FEN, respectively, Figure 3B). Similarly, estimates of Tajima’s *D* between ALP and FEN also differed, though the difference is unlikely to be biologically meaningful (mean *D* = –1.1 and –1.04, respectively, Figure 3C). Finally, because alleles at very low frequency could simply be false SNPs (*e.g.*, sequencing errors), we also calculated the summary statistics for all alleles with frequencies ≥ 0.01. Results based on this subset of 2062 SNPs gave similar estimates for both datasets: π = 0.0049 and 0.005, *D* = –1.03 and –1.03 for FEN and ALP datasets, respectively.

### Population divergence

Population divergence between the two domains was estimated for each single SNP. We chose Hudson’s estimator of *F*_ST_ since it is less sensitive to sample size difference than other estimators (Bhatia *et al.* 2013). More than half of the SNPs had an estimate of *F*_ST_ value less than 0.01, and close to 90% of the SNPs had an estimate of *F*_ST_ below 0.1 (Figure 3D). Twenty-seven highly divergent SNPs had *F*_ST_ values above 0.3 (99% quantile). About half of these high *F*_ST_ SNPs were distributed over four genes, *PaGI* (2 SNPs), *PaAP2L3* (5), and *Pa02776N18* (5) and *Pa02728N15* (5). An overall estimate of population divergence was calculated based on combining *F*_ST_ values across all SNPs. We first averaged the numerator and denominator of Hudson’s estimator separately and used the ratio as the genome-wide estimate of *F*_ST,_ which gave a value of 0.047. The overall estimate of *F*_ST_ obtained by averaging the individual *F*_ST_ values of all SNPs gave an estimate of 0.032. This smaller value is expected since this second estimator of *F*_ST_ is more sensitive to ascertainment bias and private SNPs (Bhatia *et al.* 2013; Jackson *et al.* 2014). If allele frequency was restricted to values ≥ 0.01, *F*_ST_ was 0.037 between the two domains. So, all estimates show that population divergence between the two domains is limited. To find out whether this is due to a recent divergence, or to gene flow, we estimated migration and divergence time.

### Joint demographic history inference

We simulated the joint site frequency spectrum (SFS) based on an “Isolation with migration” model, using all silent SNPs. Three ancestral population models were tested: a constant, expanding, or bottlenecked ancestral population size (Figure 2). Demographic inference was conducted using 1000 bootstraps of the SFS, and we reported the 95% confidence interval for each parameter (Table 3 and Figure S2). Model comparison using Akaike’s weight of evidence (*w*) showed that 83.8% of bootstraps supported the constant size model (mean *w* = 0.53), while only 8.7% and 7.5% supported the bottleneck (mean *w* = 0.24) and growth models (mean *w* = 0.23), respectively. Two-dimensional multinomial comparison between the constant size model and observed data also showed a good fit with the maximum Anscombe residual less than 2.8 (Figure S3). Assuming a mutation rate of 1.1 × 10^−9^ per site per year (Chen *et al.* 2012b), and an average generation time of 25 yr, we obtained an effective population size around 41,000 (95% C.I.: 13,000–47,000) for FEN and a slightly larger one, at 51,000 for ALP (95% C.I.: 45,000–65,000). The ancestral population size was nearly ten times larger (∼400,000). As noted by Becquet and Przeworski (2009), isolation–migration models often yield an estimate of the ancestral effective population size that is much larger than that of either descendant population. Simulations indicate that this large value of the ancestral population size could reflect the fact that the ancestral population was actually structured. The two populations started to diverge around 5 million yr ago (Mya) (95% C.I.: 3.6 Mya–14 Mya, for the constant size model). We also found more migrants in the direction from north to south (M∼15 per generation, 95% C.I.: 1.7–19.5) than in the opposite direction (M < 2 per generation; 95% C.I.: 0.9–8.9).

**Table 3:** Posterior distribution modes and 95% confidence intervals of demographic parameters estimated with fastsimcoal2

Model	N_FEN_*^a^* (× 10^3^)	N_ALP_ (× 10^3^)	N_0_ (× 10^3^)	T (× 10^6^)	T_BOT_ (× 10^3^)	M12	M21	α
Constant*^b^*	41 (13–47)	51 (45–65)	397 (164–469)	5.2 (3.6–14)	NA	14.6 (1.7–19.5)	1.6 (0.9–8.9)	NA
Growth*^b^*	41 (12–46)	52 (45–66)	390 (159–470)	5.0 (3.6–16)	NA	14.9 (1.4–19.8)	1.6 (1–8.7)	3.6e-5 (6.3e-8–1.7e-3)
Bottleneck*^b^*	41 (17–48)	51 (45–65)	397 (131–474)	4.8 (1.1–14)	5 (1.1–19)	14.9 (12.1–18.9)	1.6 (0.9–7.6)	NA

See text for definition of parameters. The models are described in Figure 2. The constant ancestral population size model is the most likely. NA, not available.

a Mutation rate μ = 1.1 × 10^−9^ per site per year and a generation time = 25 yr.

b Mode and 95% confidence interval are reported for each parameter.

### SNP divergence analyses based on F_ST_ and AFD

**Figure 4:**
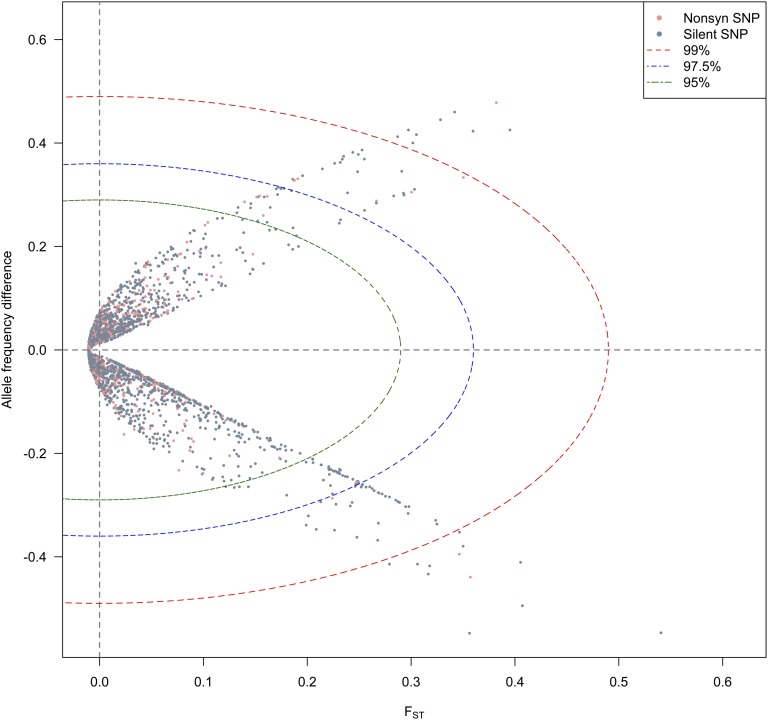
Scatterplot of divergence analysis based on *F*_ST_ and allele frequency difference. Gray dots show the silent SNPs, and pink dots show the nonsynonymous SNPs. Divergence distance of SNP based on *F*_ST_ and AFD. Cutoff envelopes for different significance levels are shown in colored ellipses.

*F*_ST_ is a fixation index and measures genetic divergence between populations, but is not an absolute measure of population differentiation: two populations can have no alleles in common and yet have a very low *F*_ST_ value, if each of them is highly variable (Charlesworth 1998). Hence, part of the information present in the difference in allele frequencies between populations is not captured by *F*_ST_. We therefore calculated a new summary statistic div*D* from both AFD and *F*_ST_ values (see *Materials and Methods*). While over half of the SNPs with AFD < 0.1 and *F_ST_* < 0.01 clustered tightly around the origin, SNPs spread quickly as AFD increased (Figure 4). We also separated the SNPs into nonsynonymous and silent sites (Figure S4). The nonsynonymous SNPs in general tend to be less diverged (less spread in *F*_ST_ and AFD values) between FEN and ALP populations than silent ones. The distance distribution between these two groups of SNPs differed significantly (Kolmogorov-Smirnov test *p*-value = 1.9e–4) in both mean (0.87 and 1.1 for nonsynonymous and silent SNPs, respectively, Wilcoxon Rank Sum test *p*-value = 7e–4) and variance (0.55 and 1.1, F-test *p*-value < 2.2e–16). This difference could reflect the fact that nonsynonymous mutations are more likely to be under purifying selection and therefore exhibit much lower divergence between geographically isolated populations than silent mutations that are primarily affected by genetic drift (Barreiro *et al.* 2008; Jackson *et al.* 2014; Maruki *et al.* 2012).

On the other hand, SNPs involved in local adaptation may show higher divergence level due to differences in environmental stress across populations. To control for the effect of genetic drift, we took advantage of the simulated SFS to estimate the variation in *F*_ST_ and AFD that could be generated by the demographic history. We then built significance envelopes at 5%, 2.5%, and 1% to select SNP outliers based on their ranked distance to the origin (see details in *Materials and Methods*). This resulted in 132, 68, and 15 SNPs in the 5%, 2.5%, and 1% significance level tails, respectively (see the detailed list in File S1). Remarkably, the majority (> 70%) of these outliers came from nine genes: *PaAP2L3* (39), *PaPRR3* (12), *Pa02728N15* (13), *Pa02745J16* (11), *Pa02776N18* (10), *Pa0273A22* (8), *PaGI* (3), *Pa00931A07* (3), and *Pa02749P05* (3). Another interesting observation is that there were 68 outlier SNPs (5% tail), where the allele frequencies in the northern populations (FEN) were close to fixation or loss (freq ≥ 0.99 or freq ≤ 0.01, 30 were found within the 2.5% significance tail, and four were found within the 1% significance tail). This was most obvious for the gene *PaAP2L3*, which showed a clear enrichment in such SNP (35 SNPs). In contrast, only two SNPs were found in the southern populations (ALP), with frequencies close to 0, and none close to fixation, while this ratio was 518/269 in the total dataset. On the other hand, we also noticed that more outlier loci in the ALP populations had intermediate allele frequencies (0.3–0.7) than in FEN (55 *vs.* 21 at 5% tail, 40 *vs.* 9 at 2.5% tail, 13 *vs.* 1 at 1% tail, but 357 *vs.* 303 in the total dataset). The enrichment of fixed/lost alleles, and lack of intermediate alleles in northern populations, could indicate a stronger purifying selection pressure in northern populations than in southern ones, if one assumes that the two domains had similar demographic histories. Alternatively, Fennoscandian populations were much more affected than Alpine ones by glacial cycles during the Quaternary, and the difference could also be the result of processes such as loss of heterozygosity or allele surfing during postglacial range expansion (*e.g.*, Goodsman *et al.* 2014, and references therein).

By using the same strategy as for individual SNPs, we calculated div*D* for genes by averaging values of SNPs, and selected outlier genes as genes outside the simulated 95% envelope. Seven genes showed a higher average level of divergence including *PaAP2L3*, *PaGI*, *PaEBS*, *PaARFL*, *PaAtCUL3a*, *Pa02717F24*, and *Pa02728N15*.

To account for the difference between synonymous and silent SNPs, we also applied FAMD, which incorporated the nonsynonymous/silent information as a categorical variable. The first three components explained 41%, 32% and 27% of the variance, respectively (Figure S4). We observed 126 outlier SNPs for separate div*D* analyses, and 125 outlier SNPs for the FAMD analysis. No additional outliers were identified by either method compared to the analysis of mixed silent and nonsynonymous SNPs (File S1).

Low frequency alleles could still be sequencing errors even though we performed cross-validation among replicates and filtered the data carefully during SNP calling (see *Materials and Methods*). Thus, we also assessed the robustness of our results by redoing the analyses in subsets of data with minor allele frequency ≥ 0.01 and 0.1. The subsets included 2082 and 963 SNPs, respectively. When only SNPs with minor allele frequency ≥ 0.01 are retained, we obtained 94 outliers at 5% tail, 52 at 2.5% tail, and 20 at 1% tail, 89, 49, and 12 of which overlapped with those identified in the total dataset, respectively. When the threshold was at frequency ≥ 0.1, we identified 39 outliers in the 5% tail, 15 at 2.5%, and 2 at 1%. All of them had been reported in the total dataset. The distance distributions of the three datasets [all SNPs (full spectrum), minor allele frequency ≥ 0.01, and minor allele frequency ≥ 0.1] were highly correlated (Pearson’s correlation test, *p*-value < 2e–16, coefficient = 0.99). The relative proportions of outliers selected at 5% remained similar in all three datasets (4–4.7%). Compared to the complete dataset, a decrease of the number of outliers was observed in the most stringent subset (frequency ≥ 0.1) at 2.5% tail (from 2.4 to 1.56%), and at 1% tail (from 0.5 to 0.2%).

### Testing for local adaptation: F_ST_ outliers

**Figure 5:**
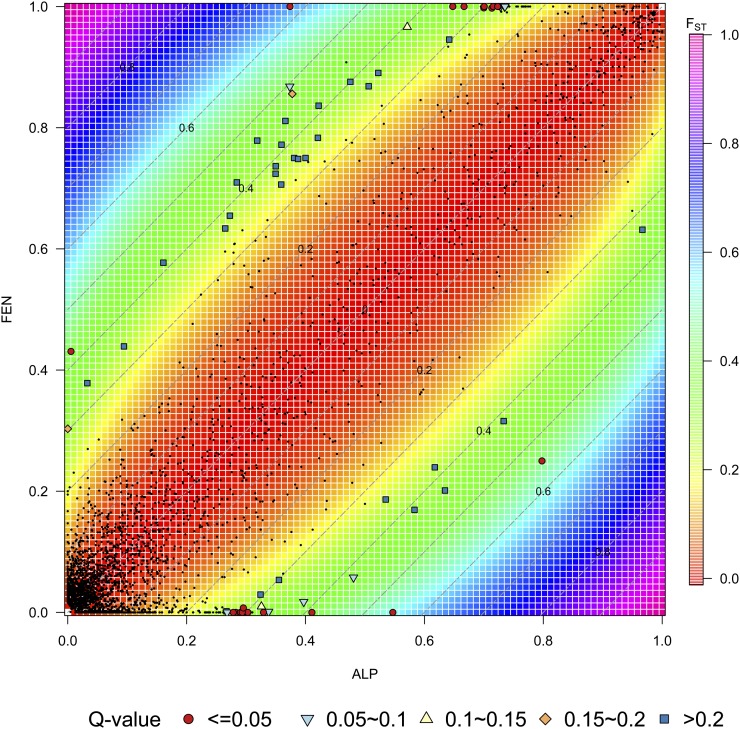
Relationship between *F*_ST_ and allele frequency difference. The color strips show the *F*_ST_ contours, and the gray dash lines parallel to the diagonal show the AFD contour lines. SNPs used in this study were mapped using points: small black dots are SNPs with divergence distance under 97.5% tail of simulated distribution, while outliers are shown in difference shapes, and colors corresponding to their q-values calculated by BayeScan. The red dots correspond to outliers for *F*_ST_ values, and allele frequencies that were also outliers with BayeScan with Q-value ≤ 0.05. The analysis based on *F*_ST_ and allele frequency difference also picks outliers with BayeScan Q-value ranging from 0.05 to 0.8.

To identify candidate SNPs under local adaptation, we used BayeScan v2.1 (Foll and Gaggiotti 2008). The method decomposes *F*_ST_ into a population-specific effect shared by all SNPs, and a locus-specific effect shared by all populations. The presence of selection is inferred if the locus-specific effect is necessary to explain the observed divergence. The log probabilities of choosing alternative models were calculated and corrected for multiple tests as Q-values (Figure 5). By assuming a prior odd of neutral to selected mutations at 10, we identified 24 outlier SNPs at Q-value ≤ 0.05 containing four nonsynonymous (*PaGI_F4_233*, *PaPRR3_2066*, *PaAP2L3_1420*, and *Pa0279G15_694*) and one synonymous (*PaAP2L3_1621*) SNP. Outliers came mainly from *PaAP2L3* (11), *Pa02728N15* (4), and *Pa0273A22* (3), but also *PaGI* (2), *PaPRR3* (1), *PaAtCUL3a* (1), *Pa02711F10* (1), and *Pa0279G15* (1). All 24 outliers at the 5% cut-off of Q-values had been also identified by div*D* analyses at 2.5% significant tail. Only four of them were nonsynonymous changes. If we chose a Q-value cutoff at 1%, six outliers would be identified, and four of them were also picked by div*D* at 1% significant tail (File S1).

## Discussion

In previous studies, we have detected departures from neutrality among phenology-related genes using scattered samples across the whole range of Norway spruce (Källman *et al.* 2014), and evidence of local adaptation by analyzing variation along latitudinal clines in Norway spruce and Siberian spruce (Chen *et al.* 2012a, 2014). In the present study, we used an intermediate sampling strategy as well as a different sequencing approach, and sequenced 88 genes in two pools of individuals drawn from the two main historical domains of Norway spruce: the Alpine and Fennoscandian domains. We then used silent polymorphisms to infer the demographic history of the two domains, and all polymorphic sites to test for the presence of selection. Different approaches, including a novel one, were used for the latter. The main conclusions are that, based on the silent variation in this sample of 88 genes, the two domains diverged some 5 Mya and that purifying and positive selection both played an important role in the divergence of the two domains. In particular, we confirmed the importance of selection at a large geographic scale on genes related to the circadian clock, such as *PaPRR3* and *PaGI*. Below we shall discuss those results in turn, but we start by addressing some of the technical issues encountered when analyzing pooled sequence data.

### Estimate population genetic parameters from pooled ngs data

Pooled sequencing technology has proven to be not only cost-effective but, more importantly, a fast and reliable access to estimates of the site frequency spectrum and population divergence if a proper sampling and statistical strategy is used (*e.g.*, Boitard *et al.* 2012; Futschik and Schlotterer 2010; Lynch *et al.* 2014; Fracassetti *et al.* 2015). To minimize the error rate in this study, we took a number of precautionary steps at both the data collection and analysis phases of the project. We first sequenced both pools twice in order to increase the detection power of rare alleles, while securing a lower false positive rate (Bansal 2010). Since the distribution of short-read coverage could be a major source of error in estimation of allele frequency and population divergence (Futschik and Schlotterer 2010; Lynch *et al.* 2014), we then applied a two-step down-sampling to minimize the unequal coverage across libraries and genes, as well as different regions of the amplicons. Finally, subsampling at frequencies ≥ 0.01 and ≥ 0.1 indicates that estimates of polymorphism and divergence are not very sensitive to rare alleles. Although it is hard to estimate the accuracy of allele frequency estimates, congruence with previous studies lends support for the pooled approach. Earlier estimates of multi-locus nucleotide diversity, Tajima’s *D* and *F*_ST_ in *P. abies* populations are of the same order of magnitude (π = 0.0038, *D* = –0.90 (Chen *et al.* 2010); *F*_ST_ = 0.035–0.05 between populations of 61–68°N and populations of 47–53°N (Chen *et al.* 2012a); mean π = 0.0021, *D* = –0.92, *F*_ST_ ∼0.12 in comparisons between populations of 66°N and populations of 46–47°N (Heuertz *et al.* 2006) as the ones obtained in this study (π = 0.005, *D* = –1.0, and *F*_ST_ = 0.047). The smaller nucleotide diversity and larger *F*_ST_ estimates obtained in Heuertz *et al.* (2006) could simply be due to the difference in sample sizes and choice of genes, since the present study and studies that are of more similar sizes (Chen *et al.* 2012a; Larsson *et al.* 2013) gave highly similar results.

### Population divergence history

According to the analysis of the joint frequency spectrum of synonymous sites, the two domains diverged around 5 Mya, and gene flow between them has been extensive and mostly in a north to south direction. This divergence estimate is much larger than that proposed earlier by Lagercrantz and Ryman (1990) who, based on Nei’s genetic distance calculated from 22 allozyme loci, obtained an approximate divergence time of 40,000 yr. Our estimate of 5 Mya, however, is of the same order of magnitude as the 6 million yr obtained by Lockwood *et al.* (2013) from plastid (matK, trnk, and rbcL), mitochondrial (nad1 and nad5), and nuclear (4CL) sequences. Given that the latter primarily reflects divergence at cytoplasmic loci, one would expect to obtain shorter estimates of divergence since the effective population sizes are twice as large for nuclear as for cytoplasmic markers in monoecious species. On the other hand, neither Lockwood *et al.* (2013) nor Lagercrantz and Ryman (1990) considered migration when estimating divergence time, and hence their estimates and that obtained here are difficult to compare. Finally, one major caveat of the present estimate is the fact that synonymous sites come from genes that might have been under selection, and thereby could have been affected by nearby selection on nonsynonymous sites. Although we could not estimate linkage disequilibrium in these pooled data, a rapid decay of LD within genes has generally been found in previous studies (Heuertz *et al.* 2006; Larsson *et al.* 2013), so polymorphism at the silent sites is unlikely to have been strongly affected by selection across the 88 genes. Also, when using Bayescan, a single synonymous SNP was detected. So, altogether, linked selection is unlikely to have had a major impact on demographic inferences.

### Evidence of selection

Previous studies of genetic differentiation have focused either on quantifying population divergence and past demography from variation at neutral sites, or on detecting genomic region under diversifying selection by using genome scans. Despite overwhelming evidence of its importance in evolution, the effect of purifying selection on population divergence has seldom been considered explicitly. Recently, Jackson *et al.* (2014) compared genetic differentiation between French and Rwandan populations of *Drosophila melanogaster* at 0-fold sites, four-fold sites and introns. They showed that, in agreement with the expected effect of purifying selection, sites in 0-fold sites in conserved genes are less differentiated than those in less conserved genes. In the present study, the nonsynonymous SNPs tend to have both lower frequency differences, and lower *F*_ST_ values between the two domains than silent ones. Assuming that both synonymous and nonsynonymous sites are otherwise affected in the same way by demography, purifying selection seems the most likely explanation to this observation. We note that this does not contradict the enrichment in nonsynonymous outliers in sites with high *F*_ST_. These outliers are, by definition, a very small proportion of all sites, and here we have been looking at the overall effect of divergence on those sites. Using Bayescan, we also detected a handful of outliers. Figure 5 illustrates the difference between the outliers detected by the divergence analysis, and those detected by Bayescan. In our specific case, Bayescan preferentially picks outliers on the edges of the SFS matrix (where alleles are close to fixation or loss in one of the two populations), while the divergence analysis retains SNPs over a larger spectrum of frequencies in both populations. Bayescan’s outlier SNPs have higher *F*_ST_ values than SNPs with the same allele frequency difference (AFD) between populations but that are far from fixation in either populations (SNPs with the same AFD are distributed along the gray dashed lines in Figure 5). However, this also means that given SNPs of same *F*_ST_ values (located within the same color strip in Figure 5) Bayescan outliers have lower AFD values (since *F*_ST_ contours are bent, and are not parallel with AFD lines except near the diagonal). Few SNPs with Q-value lower than 0.2 in Bayescan are in nonmarginal areas, and only one with intermediate frequencies has a Q-value lower than 0.05. Therefore, in this study, and perhaps more generally, Bayescan tends to target old mutations that went to fixation or new mutations that have been under strong selective sweep. For the genomic areas where these loci are located, migration barriers may have long been established, even if the average migration rate across all loci has been high between the two domains of Norway spruce. However, this is not always the case, and, for loci under weaker selection, for example, loci underlying a quantitative trait controlled by a large number of loci, each of which is weakly affected by selection (Latta 1998; Le Corre and Kremer 2012), gene flow will keep introducing new variants and prevent the locus to go to fixation. In such cases, AFD would be high, but neither allele will be fixed (lower-right or upper-left areas of Figure 5). These loci will be identified by the divergence distance method as putatively under selection, but not by Bayescan, which is more stringent. Additionally for pooled samples, to be able to select Bayescan outliers large sample sizes are required as the outliers usually have a frequency close to fixation or loss in one of the populations (Lynch *et al.* 2014). Many outliers of our divergence analysis have intermediate frequency, and thus can lower the requirement for the number of individuals. Finally, one caveat is that, as for many outlier methods, the present method can lead to false positives and false negatives if the wrong demographic model is used it the analysis.

The two most interesting loci detected in the present study are the circadian clock genes *PaGI* (*Gigantea*) and *PaPRR3*, since they were also identified as top candidate genes for local adaptation in previous studies. Gigantea homologs consistently show evidence of non-neutral evolution, both in conifers (Chen *et al.* 2012a, 2014; Holliday *et al.* 2010), and in angiosperms (Keller *et al.* 2012; Olson *et al.* 2013). Its pattern of polymorphism within spruce species is intriguing, but has been hard to analyze because of the lack of silent polymorphism in the parts of the gene that were sequenced so far. Repeated selective sweeps have been proposed for *Populus balsamifera*, where a similar pattern of polymorphism is observed (Keller *et al.* 2012), but this hypothesis remains to be confirmed. *PaPRR3* has not been included as often in candidate gene panels, and less is known about it. In Norway spruce, *PaPRR3* departed from neutrality when departure from neutrality was tested from a range-wide sample (Källman *et al.* 2014), but *PaPRR3* did not exhibit latitudinal cline in allele frequency as one might have expected from a circadian clock gene if it were directly involved in adaptation to local conditions (Chen *et al.* 2012a). Finally, it is also worth mentioning *AP2L3* as a gene of special interest. While genetic variation in this gene has not been studied as extensively as that of *GI*, *PRR3*, or *FTL2*, *AP2L3* exhibits an intriguing pattern in the present study, and its involvement in development in Norway spruce (Nilsson *et al.* 2007) makes it an interesting candidate gene.

In conclusion, this study shows that combining PCR with a pooled sequencing strategy is a cost-effective and reliable approach for generating multilocus population genetic data sets in nonmodel organisms. The inferred pattern of diversity and population divergence clearly shows that the Alpine and Fennoscandian domains of Norway spruce remain genetically closely related although they diverged 5 Mya. This is probably due to pollen flow, as the two domains are clearly differentiated for mitochondrial DNA, which is maternally inherited (Lockwood *et al.* 2013; Tsuda *et al.* 2016). In addition we detected a set of genes deviating from neutral expectations. Together with earlier studies of candidate genes for adaptation to local light conditions, both *PaPRR3* and *PaGI* are emerging as central genes that would certainly deserve further attention. In particular, it would be very informative to resequence a larger part of the PaGI gene in a larger sample in order to test for selection, and to conduct more extensive functional studies in both genes.

## Supplementary Material

Supplemental Material
