# Supplementary material for: Identifying Genetic Signatures of Natural Selection Using Pooled Population Sequencing in Picea abies
Source: G3 (Bethesda). 2016 May 2;6(7):1979–89. doi: 10.1534/g3.116.028753 (PMC4938651; doi:10.1534/g3.116.028753)
Supplement: Supplemental Material [file supp_6_7_1979__index.html]

Identifying Genetic Signatures of Natural Selection Using Pooled Population Sequencing in Picea abies — Supplemental Material 

# Identifying Genetic Signatures of Natural Selection Using Pooled Population Sequencing in *Picea abies*

## Supplemental Material for Chen *et al.*, 2016

**Files in this Data Supplement:**

- Figure S1 - Mean short-read coverage distributions along the whole gene body. (.pdf, 217 KB)
- Figure S2 - Marginal distribution of estimated parameters by fastsimcoal2 under (A) constant size model (B) population growth model (C) bottleneck model. (.pdf, 400 KB)
- Figure S3 - Comparison of two-dimensional site frequency spectra inferred by fastsimcoal2 with that of observed data. (.pdf, 266 KB)
- Figure S4 - Divergence distance of non-synonymous and silent SNPs based on FAMD. (.pdf, 338 KB)
- File S1 - Description of the outlier SNPs. (.xlsx, 50 KB)
