# Supplementary figures and images for: Identifying Genetic Signatures of Natural Selection Using Pooled Population Sequencing in Picea abies
Source: G3 (Bethesda). 2016 May 2;6(7):1979–89. doi: 10.1534/g3.116.028753 (PMC4938651; doi:10.1534/g3.116.028753)

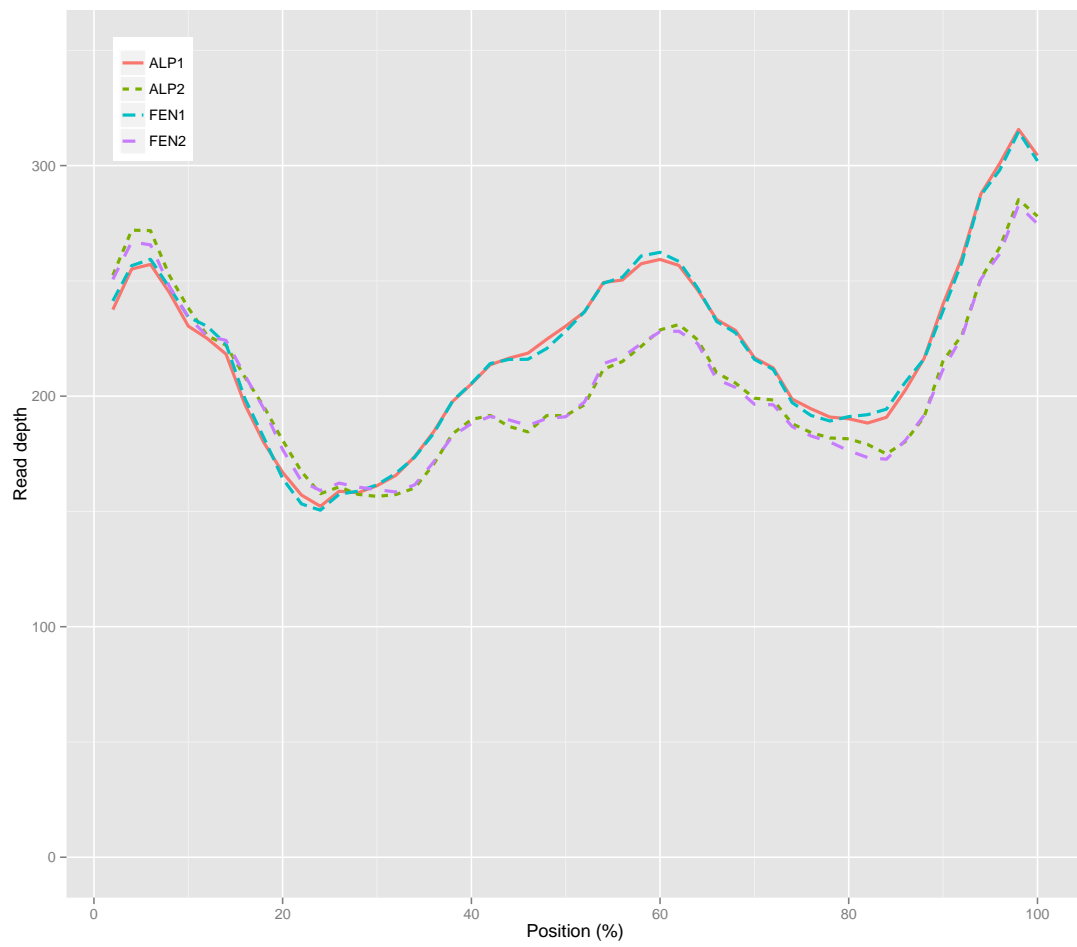

**Figure S1. Mean short-read coverage distributions along the whole gene body.**

Supplement: Supplemental Material [file supp_g3.116.028753_FigureS1.pdf]
