# Supplementary material for: Identifying Genetic Signatures of Natural Selection Using Pooled Population Sequencing in Picea abies
Source: G3 (Bethesda). 2016 May 2;6(7):1979–89. doi: 10.1534/g3.116.028753 (PMC4938651; doi:10.1534/g3.116.028753)
Supplement: Supplemental Material [file supp_g3.116.028753_FigureS2.pdf]

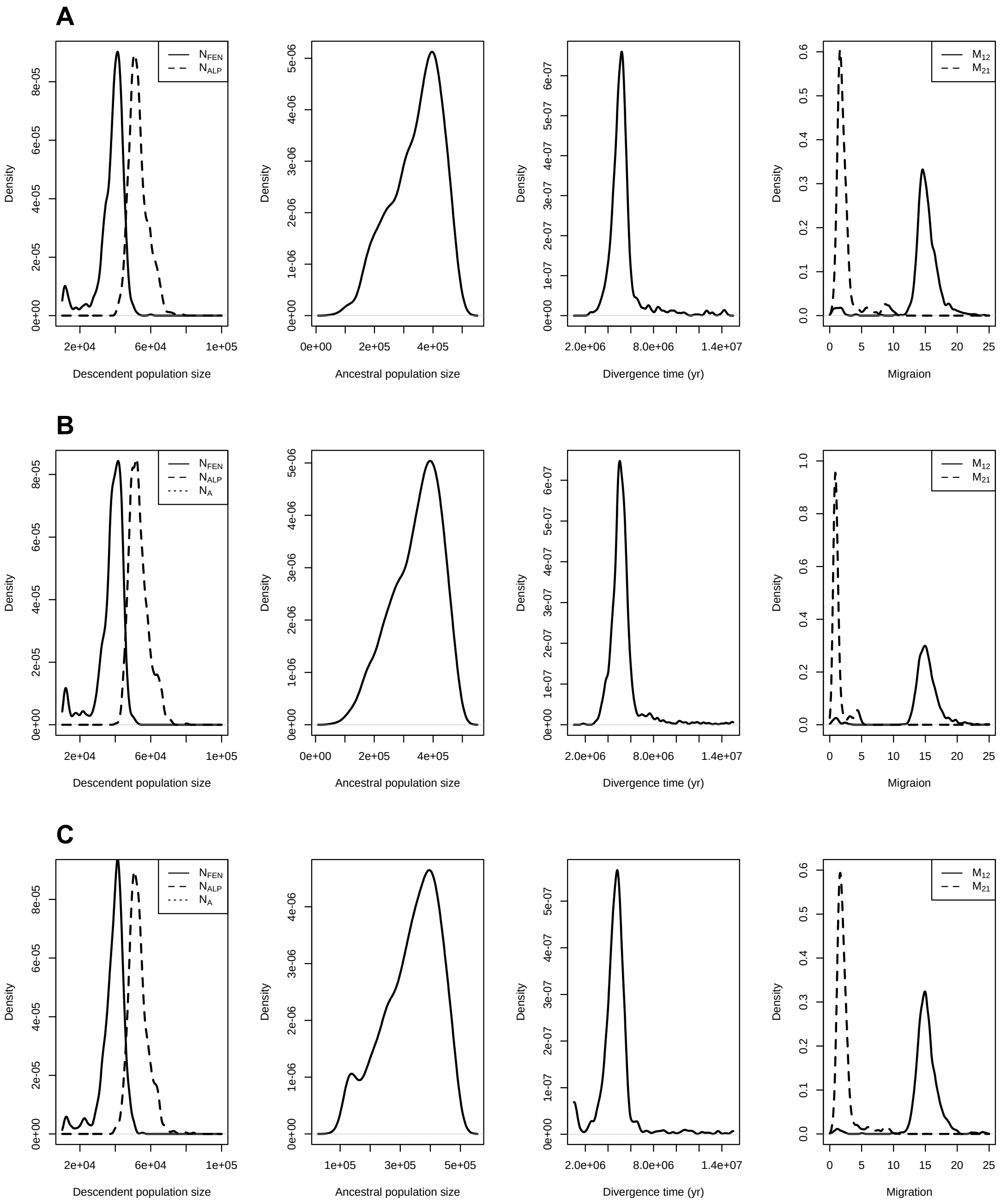

Figure S2. Marginal distribution of estimated parameters by fastsimcoal2 under (A) constant size model (B) population growth model (C) bottleneck model. M_{12} represents the migration from FEN to ALP and M_{21} from ALP to FEN.
