# Supplementary material for: Identifying Genetic Signatures of Natural Selection Using Pooled Population Sequencing in Picea abies
Source: G3 (Bethesda). 2016 May 2;6(7):1979–89. doi: 10.1534/g3.116.028753 (PMC4938651; doi:10.1534/g3.116.028753)
Supplement: Supplemental Material [file supp_g3.116.028753_FigureS3.pdf]

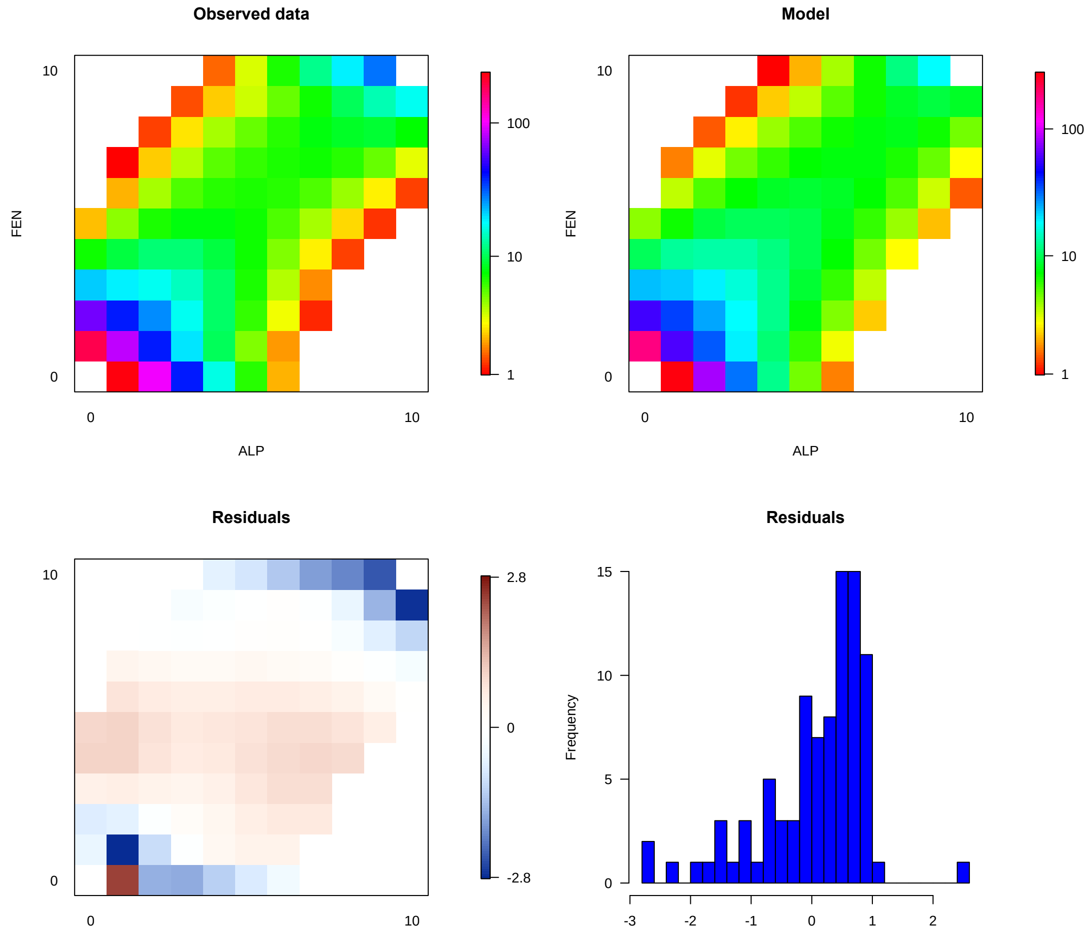

Figure S3. Comparison of two-dimensional site frequency spectra inferred by fastsimcoal2 with that of observed data. Allele frequencies are projected into a 10×10 grid. Multinomial comparison was applied by computing Anscombe residuals.
