# Supplementary material for: Identifying Genetic Signatures of Natural Selection Using Pooled Population Sequencing in Picea abies
Source: G3 (Bethesda). 2016 May 2;6(7):1979–89. doi: 10.1534/g3.116.028753 (PMC4938651; doi:10.1534/g3.116.028753)
Supplement: Supplemental Material [file supp_g3.116.028753_FigureS4.pdf]

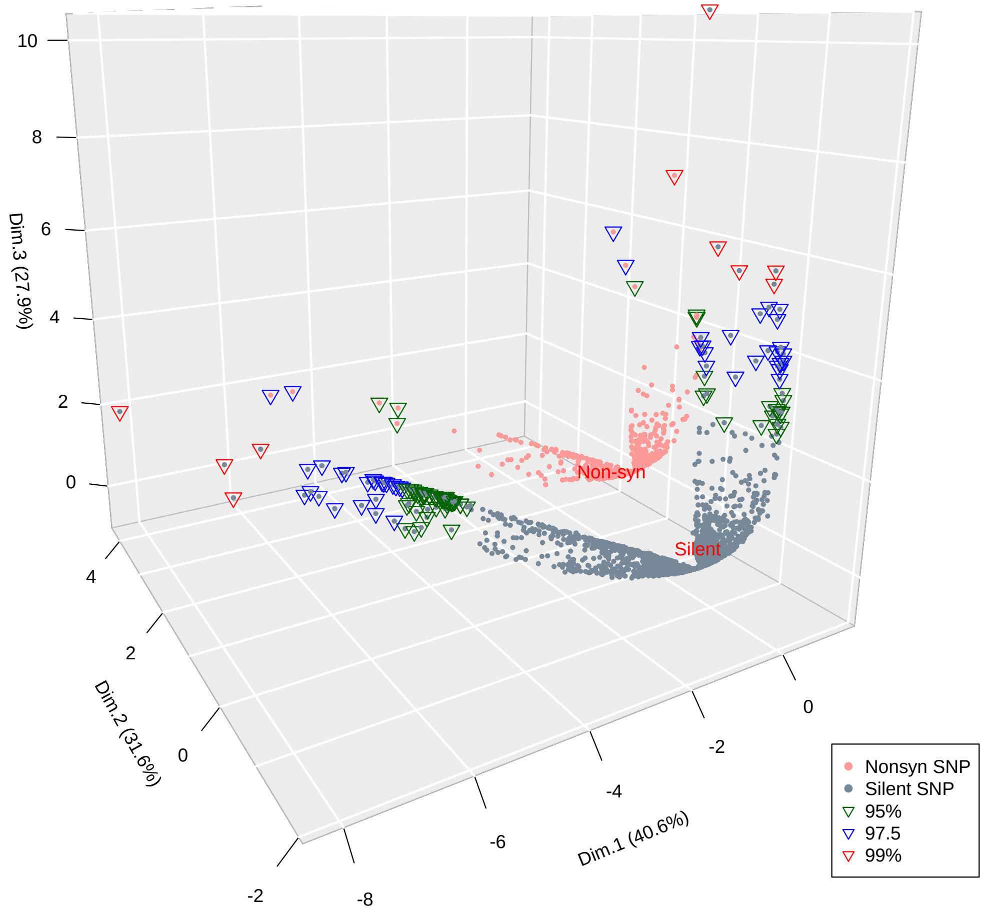

Figure 4S Divergence distance of non-synonymous and silent SNPs based on FAMD. Outlier SNPs are highlighted with triangles. Colors of triangle show the three cutoffs.
